# Effects of the COVID-19 pandemic on delays in diagnosis-to-treatment initiation for breast cancer in Brazil: a nationwide study

**DOI:** 10.3332/ecancer.2023.1570

**Published:** 2023-07-07

**Authors:** João Henrique Fonseca do Nascimento, Cleonice Nascimento da Silva, André Gusmão-Cunha, Marinho Marques Silva Neto, André Bouzas de Andrade

**Affiliations:** 1 Life Sciences Department, Universidade do Estado da Bahia (UNEB), Salvador 41150-000, Brazil; 2 Anesthesiology and Surgery Department, Universidade Federal da Bahia (UFBA), Salvador 40026-010, Brazil; 3 Hospital Santa Izabel (HSI), Bahia Cancer Institute, Salvador 40050-410, Brazil; a https://orcid.org/0000-0001-8750-6116; b https://orcid.org/0000-0002-8356-6426; c https://orcid.org/0000-0001-7762-168X; d https://orcid.org/0000-0002-9728-7268; e https://orcid.org/0000-0002-4010-0415

**Keywords:** breast cancer, COVID-19, time-to-treatment, treatment delay

## Abstract

**Background:**

Short period from diagnosis to breast cancer (BC) treatment initiation remains challenging for the public health system in Brazil, which may have been further affected by the coronavirus disease-2019 (COVID-19) pandemic. This study assessed BC diagnosis-to-treatment intervals (DTi) in Brazil and the possible effects of the COVID-19 outbreak on delays.

**Methods:**

The Painel de Monitoramento de Tratamento Oncológico database was queried to obtain the number of Brazilian patients with a BC confirmed diagnosis and initiating cancer treatment in the pre-COVID-19 (2013–2019) and during the COVID-19 (2020–2021) periods, adopting a 60-day limit as timely treatment. A *p*-value of <0.05 was considered significant.

**Results:**

A total of 315,951 cases were included (females: 99.3% and males: 0.7%), of which 251,667 and 64,284 records were computed before and during the COVID-19 years, respectively. Most patients failed to perform the first cancer treatment within 60 days (>60: 51.8%). We observed an upward trend in the number of BC treatments provided in the pre-COVID-19 years (*r*^2^ = 0.9575; *p* < 0.05), but the volume of treatments exhibited an average reduction of 24.6% yearly during the COVID-19 pandemic. The average DTi in days was 122.4, 122.5 and 122.3 in the total period studied, before and during the COVID-19 outbreak, respectively. The arrival of COVID-19 in Brazil increased the chances of treatment delay (OR = 1.043; *p* < 0.05) and inverted the proportion of early/advanced stages at BC diagnosis (55.8%/44.2%–48.4%/51.6%).

**Conclusion:**

COVID-19 has imposed changes in BC care in Brazil, reducing the number of treatments provided by the Brazilian public health system, increasing the chances of delayed treatment initiation despite no differences in DTi averages being identified, and raising the proportion of advanced-stage diagnoses.

## Background

Breast cancer (BC) is the most frequent malignant neoplasm in Brazilian women, excluding non-melanoma skin cancer, and also figures as the first cause of female death by cancer in the country [1–3]. In Brazil, 66,280 new BC cases were estimated for 2022, accounting for nearly a third of all malignancies and corresponding to an adjusted incidence rate of 43.74 cases per 100,000 Brazilian women [1, 2, 4]. BC also affects men, yet rarely, representing about 1% of all cases [1, 5].

BC can be curable in up to 80% of patients when detected early and treatment started promptly [6–9]. The period from diagnosis confirmation to treatment initiation is the time interval that guidelines in oncology typically recommend minimising for better outcomes and overall survival, and, in this perspective, the Brazilian Government decreed Federal Law number 12,732 in November 2012, also known as the 60-Day Law, in an attempt to decrease treatment delays and its consequences subsequently [3, 9–11]. This legislation dictates the maximum interval of 60 days that a patient with any cancer has to wait to initiate his/her oncological treatment, counted from the diagnostic confirmation of the malignant neoplasm by histopathological analysis [3, 10, 11]. The law was reinforced and came into effect in 2013, when the Brazilian Ministry of Health instituted the National Policy for Cancer Prevention and Control through Ordinance No. 874/2013, within the scope of the Unified Health System (Sistema Único de Saúde – SUS) [10, 12].

Management based on intervals shorter than 60 days for cancer treatment onset after diagnostic confirmation remains challenging in Brazil, which could be affected by the current coronavirus disease-2019 (COVID-19) pandemic [3, 10, 13, 14]. Therefore, this population-based study aims to assess the waiting time between BC diagnosis and treatment initiation from the effective year of the 60-Day Law implementation and to evaluate the possible effects of the COVID-19 outbreak on delays in starting BC treatment in Brazil.

## Methods

This retrospective, observational and nationwide study, in time series, evaluated the impacts of the COVID-19 pandemic on diagnosis-to-treatment initiation intervals, staging and therapeutic approaches for BC in Brazil. This investigation was conducted with secondary data from a government database. Data on the initiation of oncological treatment regarding Brazilian patients with BC were obtained at the Oncology Treatment Monitoring Panel (Painel de Monitoramento de Tratamento Oncológico – PAINEL-Oncologia), linked to the Unified Health System Department of Informatics (DATASUS) platform (available at <http://tabnet.datasus.gov.br/cgi/dhdat.exe?PAINEL_ONCO/PAINEL_ONCOLOGIABR.def>). This public domain source is a free-access and online database that gathers most information on the time interval for cancer treatment onset after a confirmed diagnosis. The PAINEL-Oncologia is a nationwide and population-based database that assembles patient data from the Ambulatory Information System (Sistema de Informações Ambulatoriais) – through the Individualised Outpatient Production Bulletin (Boletim de Produção Ambulatorial – Individualizado) and the High Complexity Procedure Authorisation (Autorização de Procedimento de Alta Complexidade) form, the Hospital Information System (Sistema de Informações Hospitalares) and the Cancer Information System (Sistema de Informações de Câncer). All these information and notification forms are reported by the State and Municipal Health Secretariats through the records of the patients’ national health card (CNS) in the SUS register, and managed by the Ministry of Health. Furthermore, the PAINEL-Oncologia platform serves as a public tool for monitoring compliance with Brazilian Federal Law No. 12,732/2012. Given this, a relevant delay in time to treatment initiation for this study was defined as more than 60 days.

The data were retrieved based on the 10th Revision of the International Disease Classification (ICD-10), using the C.50 code, recorded on DATASUS as ‘Malignant neoplasm of the breast’; therefore, cases of carcinoma *in situ* were not included. The following variables were analysed: the total and the annual number of BC cases, the diagnosis-to-treatment interval (DTi) in days, the number of patients with first cancer treatment performed in 60 days or less (≤60) and more than 60 days (>60), regions of Brazil (North, Northeast, Midwest, Southeast and South), age, gender, treatment modalities (surgery, chemotherapy, radiotherapy and other) and staging (I, II, III and IV). Clinical staging of BC was reported by health professionals and services following the Malignant Tumour Classification System used by the American Joint Committee on Cancer (AJCC). When information such as time to initiate treatment, modality of treatment, staging or age was marked as ‘unknown’ or ‘missing data’, this case was not counted during the statistical analysis. The study period ranged from January 2013 to December 2021, which encompassed the pre-COVID-19 years (2013–2019) and the first 2 years of the COVID-19 pandemic (2020–2021) in Brazil. Although the COVID-19 pandemic was only declared in March 2020, the PAINEL-Oncologia platform provides year-by-year data and therefore cases in January and February 2020 (pre-COVID-19 months) were included in that respective year since the data retrieved from 2020 comprised the number of cases for the full year.

The distribution of the variables was assessed by the Shapiro-Wilk test and the *Q*–*Q* plot. The Levene’s test for equality of variances assessed the homogeneity of variances. Descriptive statistics such as average, standard deviation (±SD), median, interquartile range (IQR) with 25th (Q1) and 75th (Q3) percentiles, odds ratio (OR) and confidence intervals (CI) were used to describe the numbers and proportions, applying a chi-square test and a Fisher’s exact test to perform the data analyses. Depending on the normality of the variable distribution, a nonparametric Mann-Whitney test and a Student’s *T*-test were used to compare differences between groups. The level of significance was set at 5% (two-sided *p*-value < 0.05). Prais-Winsten linear regression [15] (*Y* = *a* + *bX*) was performed to evaluate the temporal trends and to provide the adjusted *r*^2^-values, adopting a confidence interval of 95%. To assess stability, growth or decrease of numbers over the years, the percentage change was calculated using the formula: ((next year's value − previous year's value)/previous year's value) × 100. The PAST software (Øyvind Hammer, UIO, v. 4.03) and the R software (RStudio, Inc., v. 4.0.3) were used to perform the statistical analyses.

The approval of the Ethics Committee in Research is waived since the secondary data were obtained from an online, public domain and governmental source, without identification of patients, following the current criteria of the guidelines in Resolution no. 510/2016 (07 April 2016) of the Brazilian National Council of Health, and as stated by the National Commission of Research Ethics in Brazil (available at <http://conselho.saude.gov.br/web_comissoes/conep/index.html>).

## Results

**Figure 1.**
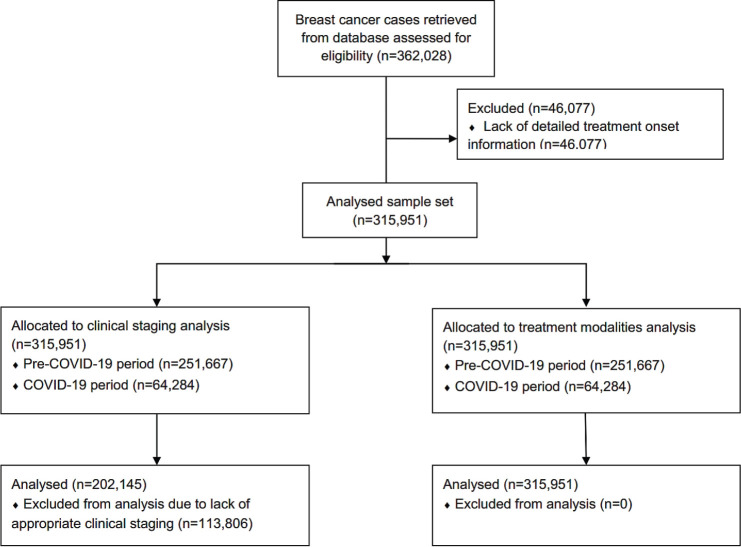
Patient flow diagram for analyses of clinical staging and treatment modalities.

In Brazil, 362,028 BC treatments were initiated from 2013 to 2021. After preliminary screening, 46,077 cases were excluded due to a lack of detailed treatment onset information, resulting in a set of 315,951 cases (Figure 1) (females = 313,825; 99.3% and males = 2,126; 0.7%). From the total, 251,667 treatments were initiated in the pre-COVID-19 years (average = 35,952/year), while 64,284 BC treatment records were computed during the COVID-19 outbreak (average = 32,142/year). The majority of patients failed to initiate BC treatment within the mandated 60-day limit after the confirmed diagnosis (≤60 = 48.2%; *n* = 152,330 versus >60 = 51.8%; *n* = 163,621) (Table 1). Excluding 2013, Figure 2 demonstrates that the annual frequency of patients with a waiting time lengthener than 60 days remained superior throughout the period.

### Regions of Brazil

**Figure 3.**
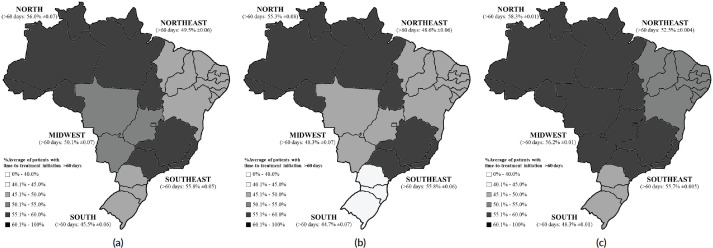
Brazilian regional differences in the average proportion of patients waiting longer than 60 days to initiate treatment (a) in the overall period (2013–2021), (b) in the pre-COVID-19 (2013–2019) and (c) during the COVID-19 (2020–2021) years.

Figure 3 exhibits the Brazilian regional differences in the average proportion of patients waiting more than 60 days to initiate their BC treatment after diagnostic confirmation, also comparing the pre-COVID-19 (Figure 3b) and the COVID-19 (Figure 3c) periods, where the average increment was more prominent in the Midwest (MW) (48.3%–56.2%; *p* < 0.05), followed by the Northeast (NE) (48.6%–52.5%; *p* < 0.05), South (S) (44.7%–48.3%; *p* < 0.05), North (N) (55.3%–58.3%; *p* < 0.05) and Southeast (SE) (55.8%–55.7%; *p* < 0.05).

### COVID-19 and trends

**Figure 4.**
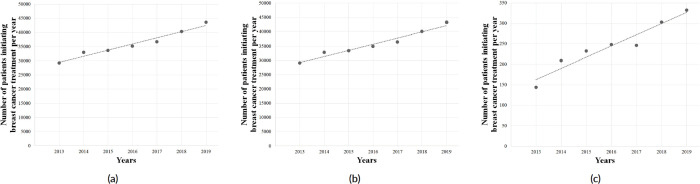
Linear regression analysis of the number of patients initiating BC (C.50) treatment in Brazil, in the pre-COVID-19 years, in the (a) total population, (b) females and (c) males. Data cover a 7-year range.

**Figure 5.**
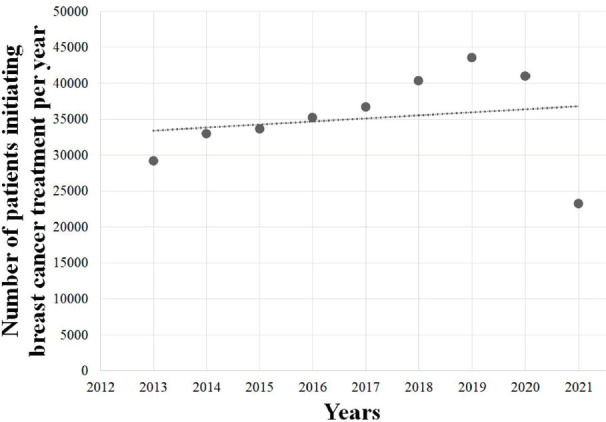
Linear regression analysis of the number of patients initiating BC (C.50) treatment in Brazil in 2013–2021. Data cover a 9-year range.

The overall period (2013–2021) exhibited no significant trend (*p* = 0.63), and no significant tendency was also observed among females (*p* = 0.64) and males (*p* = 0.42). However, the pre-COVID-19 years (2013–2019) showed an upward trend in the number of BC treatments provided by the SUS (*Y* = −4,418,731 + 2,201*X*; *r*^2^ = 0.9575; *p* < 0.05; Figure 4a), also observed among females (*Y* = −4,365,840 + 2,183*X*; *r*^2^ = 0.9574; *p* < 0.05; Figure 4b) and males (*Y* = −53,969 + 26*X*; *r*^2^ = 0.9269; *p* < 0.05; Figure 4c), with an average growth of +7.0% (±0.041), +6.9% (±0.041) and +15.8% (±0.163) per year, respectively. However, this trend was followed by an abrupt decrease during COVID-19 pandemic, with an average reduction of 24.6% (±0.26) yearly (2019–2020: −5.9%; 2020–2021: −43.3%). Similar findings were observed among females (average = −24.5%/year; SD = 0.265) and males (average = −32.9%/year; SD = 0.197) in the years 2020–2021. The regression coefficient showed that the arrival of COVID-19 in Brazil imposed an average reduction of 4,367 BC treatments provided yearly (*Y* = 39,811 − 4,367*X*), as seen in Figure 5.

**Figure 6.**
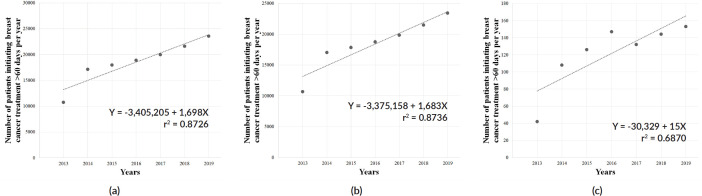
Linear regression analysis of the number of patients waiting longer than 60 days to initiate BC (C.50) treatment in Brazil, in the pre-COVID-19 years, in the (a) total population, (b) females and (c) males. Data cover a 7-year range.

**Figure 7.**
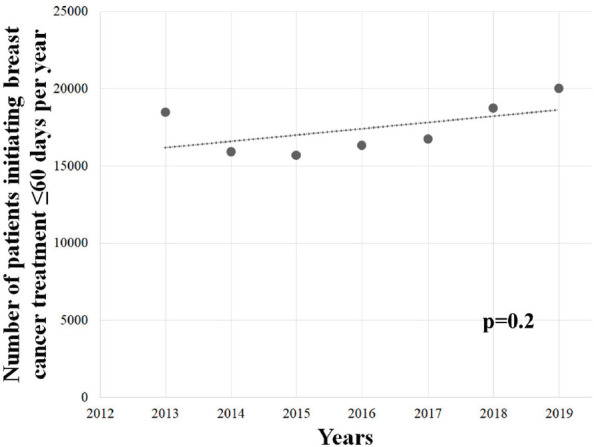
Linear regression analysis of the number of patients waiting 60 days or less to initiate BC (C.50) treatment in Brazil in the total population. Data cover a 7-year range.

Patients were associated with a greater chance of initiating BC treatment later than 60 days during COVID-19 outbreak (OR = 1.0427; 95% CI = 1.024–1.060; *p* < 0.05), and a similar association was observed among females (OR = 1.0436; 95% CI = 1.025–1.061; *p* < 0.05), but not among males (OR = 0.9092; 95% CI = 0.733–1.127; *p* = 0.41). Further, the >60 days group showed an upward trend in the pre-pandemic years (overall: *Y* = −3,405,205 + 1,698*X*; *r*^2^ = 0.8726; *p* < 0.05; Figure 6a; females: *Y* = −3,375,158 + 1,683*X*; *r*^2^ = 0.8736; *p* < 0.05; Figure 6b; males: *Y* = −30,329 + 15*X*; *r*^2^ = 0.6870; *p* < 0.05; Figure 6c) – with an average growth of +15.4% (±0.217), +15.4% (±0.216) and +32.6% (±0.618) per year, respectively – however, the set of patients in the ≤60 days group did not display similar trend (Figure 7; *p* = 0.2).

### Diagnosis-to-treatment intervals

Time intervals between diagnosis and first BC treatment varied greatly in the years 2013–2021, from patients who had a DTi of 0 days to patients who waited more than 2 years to start treatment after a confirmed BC diagnosis. Table 2 shows that the overall average DTi was 122.4 (±169.3) days yearly, and there was no difference comparing DTi before and during the COVID-19 pandemic (122.5 versus 122.3 days; *p* = 0.801). COVID-19 significantly lengthened the annual average DTi in the >60 days group (196.3 versus 203.0 days; *p* < 0.05), but no difference was observed in the group of 60 days or less (*p* = 0.422) (Table 2). Men with BC exhibited a longer average DTi than women (143.3 versus 122.3 days/year; *p* < 0.05) (Table 2). When assessing the two periods and gender, COVID-19 increased the chances of significant delays among women (OR = 1.0436; 95% CI = 1.025–1.061; *p* < 0.05), but not among men (*p* = 0.41).

### Age and gender

The overall average age was 56.6 (±15.22) years old, with a peak age and peak age group of 50 and 50–54, respectively (Table 3). Male patients were significantly older than females (63.6 versus 56.6 years old; *p* < 0.05), including in the groups of ≤60 (male = 62.3 versus female = 55.6; *p* < 0.05) and >60 days (male = 63.9 versus female = 57.2; *p* < 0.05); and also, before (male = 63.9 versus female = 56.6; *p* < 0.05) and during (male = 62.7 versus female = 56.4; *p* < 0.05) COVID-19 outbreak (Table 3). By using the peak age of 50 years as a reference, Table 4 shows that the effect of aging increased the chances of delayed treatment initiation, and this increment was more substantial during the COVID-19 pandemic.

### BC stages

As for properly staged cases (*n* = 202,145; Figure 1; Table 5), 54.04% of the general population were early cases of BC (stages I and II) and 45.96% were advanced cases (stages III and IV), and a similar proportion was observed in the pre-pandemic years (*n* = 154,380; early = 55.78% and advanced = 44.22%). However, advanced stages outnumbered the proportion of early-stage disease during the COVID-19 outbreak (*n* = 47,765; early = 48.41%; advanced = 51.59%). Moreover, with the arrival of COVID-19 in Brazil, the early-stage disease showed an average reduction in the annual diagnostic frequency (I: −11.8% and II: −2.0%), but advanced-stage disease at diagnosis increased by +30.2% for stage III and +17.5% for stage IV (Table 6). This finding is supported by the observation that COVID-19 imposed a greater overall chance of diagnosing advanced BC cases more frequently than in early stages compared to the pre-COVID-19 period (OR = 1.3441; 95% CI = 1.316–1.372; *p* < 0.05), and this association remained significant among females (OR = 1.3460; 95% CI = 1.318–1.374; *p* < 0.05) but not among males (*p* = 0.29). Beyond, COVID-19 increased the chances of detecting metastases at diagnosis in females (OR = 1.1003; 95% CI = 1.068–1.133; *p* < 0.05) but not in males (*p* = 0.2). Early stages had a greater chance of delayed treatment onset (OR = 2.2813; 95% CI = 2.240–2.323; *p* < 0.05) than advanced cases, including before (OR = 2.2895; 95% CI = 2.242–2.337; *p* < 0.05) and during the COVID-19 pandemic (OR = 2.2212; 95% CI = 2.140–2.305; *p* < 0.05).

### Modalities of treatment

Chemotherapy was the most frequently performed first BC treatment from 2013 to 2021 (60.0%), with an average of 21,062.9 (±4,051.6) treatments yearly, followed by surgery (33.1%; average = 11,613.4/year; SD = 3,238.6), and radiotherapy (6.7%; average = 2,362.0/year; SD = 450.6). Twenty thousand nine hundred thirteen (*n* = 20,913) patients were diagnosed with BC only after a non-oncological surgery, and this procedure was later counted as their first cancer treatment, which we understood as revised diagnoses, and they were excluded from the statistical analysis. As seen in Table 7, the highest percentage of patients awaited between 121 and 300 days for the first BC treatment to be performed (chemotherapy = 22.2% and radiotherapy = 35.9%), except for surgery, in which 19.6% of patients displayed a 0-day DTi. A minimal proportion of patients exhibited a 0-day interval in other treatment groups (chemotherapy = 2.9% and radiotherapy = 2.3%). The majority of patients who initiated BC treatment with surgery had the procedure performed within 60 days (≤60/>60: 57.7%/42.3%), but similar proportion was not found in other treatment modalities (chemotherapy = 41.3%/58.7% and radiotherapy = 22.4%/77.6%).

Despite exceeding the 60-day limit, surgery exhibited a significantly shorter average DTi (82.0 days) compared to chemotherapy (133.8 days; *p* < 0.05), and radiotherapy (179.4 days; *p* < 0.05) (Table 8). As seen in Table 8, significantly shorter average DTi were found when comparing surgery to other modalities in both pre-COVID-19 and during COVID-19 years. Besides, while the COVID-19 pandemic significantly shortened the average DTi for surgery (84.1–70.8 days; *p* < 0.05) and chemotherapy (134.9–130.6 days; *p* < 0.05), radiotherapy significantly lengthened the average DTi during the COVID-19 outbreak in Brazil (173.8–205.5 days; *p* < 0.05) (Table 8). Compared to surgery, all other modalities were associated with greater chances of delayed treatment onset in the years 2013–2021 (Table 9). Table 9 also shows that surgery and chemotherapy were associated with lower chances of significant delays during the COVID-19 years compared with the pre-COVID-19 period. Table 10 shows that most treatment modalities displayed a reduction in the annual volume of treatments performed during COVID-19, except for chemotherapy. The regression coefficient showed an average reduction of 5,142 BC surgeries per year with the arrival of COVID-19 in Brazil (*Y* = 18,072 − 5,142*X*; *p* < 0.05), but this finding was not observed among other therapeutic modalities (chemotherapy: *p* = 0.5 and radiotherapy: *p* = 0.07).

## Discussion

The rapid rise in the number of severe cases of COVID-19 required public policies to redistribute available hospital beds, healthcare workforce and medical equipment, which underprioritised several non-emergency medical conditions, and cancer was one of them [14, 16]. Our results showed an important reduction in the volume of BC treatments provided during the first 2 years of the pandemic in Brazil. Moreover, COVID-19 imposed a greater chance of delayed treatment onset, intensified the already significant odds of age-related delays, increased the percentage of delayed BC treatment onset across the regions of Brazil, and also worsened the delay in the group that was already starting therapy with a DTi lengthier than 60 days. Linear regression identified an upward trend in the number of patients with delayed treatment onset in the pre-COVID-19 years, highlighting that the number of patients initiating BC treatment beyond the maximum term determined by Brazilian law was increasing even before the pandemic. This observation seems to be a recurrent problem in BC care in Brazil.

A 473-patient retrospective study in Northeast Brazil between 2009 and 2011, which implies a time before both COVID-19 and the 60-Day Law, showed a median interval of 71.5 days between diagnosis and treatment onset for BC [17]. Shafaee et al. [18], by evaluating 963 BC patients in Southeast Brazil (2009–2011) showed that the average time from diagnosis to the first overall treatment and the first systemic treatment was 86.8 and 151.9 days, respectively.

The relevance of cancer treatment prompt initiation for better outcomes and lower chances of recurrence is well-established, and Brazil recognises this importance, to the point of creating a federal law regulating a medical practice of short intervals between diagnosis and cancer treatment initiation [10, 11, 13, 14]. Previous reports have shown that most BC patients still have not been receiving timely treatment in Brazil even after the announcement of the law [2, 19–21].

A 470-patient prospective study (2014–2015) conducted at the Cancer Hospital III in Rio de Janeiro/Brazil demonstrated that delayed treatment was identified in 89.1% of BC cases and the median DTi was 127 days [2]. dos Santos Andrade et al. [22], in a 304-patient study in Northeastern Brazil (2016–2019), showed that the average DTi was 62.4 days for 77.3% of cases, of which 42.0% had a delay lengthener than 90 days. In 2022, the 60-Day Law completed 10 years in force, yet our investigation evidenced that most Brazilian cases of BC still start treatment with delays of 60 days or more.

The COVID-19 outbreak appears to be associated with controversial findings regarding delays in time to BC treatment initiation worldwide, as reported by Hawrot et al. [13] in a retrospective study with 366 patients in Philadelphia/US, which, despite an 18.8% reduction in the number of new BC diagnoses, no difference was found in the average DTi comparing 2018 (44.7 days) to 2020 (44.4 days). Caswell‑Jin et al. [23] assessed 19,329,646 women and men newly diagnosed with BC in the US and showed that the proportion of patients with DTi longer than 60 days in the pre-COVID time (January/2017–March/2020: 18.9%) firstly decreased between April and May 2020 (15.7%), but then increased from June 2020 to February 2021 (19.1%). Li et al. [24] identified a lengthened DTi in 8,397 BC patients during quarantine restrictions in Hubei (3.5–7.7 days) and other provinces in China (5.7–7.7 days). On the other hand, a comparative analysis placed at the Instituto do Câncer do Estado de São Paulo/Brazil, with 268 BC patients, demonstrated that the median interval for the first cancer centre visit after breast tumour biopsy was lower during the pandemic (September/2020–January/2021: 5.4 months) than previous to it (September/2019–January/2020: 6.7 months) [25]. Similar to Hawrot et al. [13], our results showed a non-significant difference in the waiting time between the pre-COVID-19 (122.5 days) and during the COVID-19 (122.3 days) years, yet the averages were still more than double the 60-day limit recommended by Brazilian law.

One in every six Brazilian women who died of cancer in 2019 was due to BC, which emphasises the magnitude of the BC mortality burden in Brazil [5, 26]. The mantra ‘early detection saves lives’ may correlate with the tenet that late BC treatment initiation kills patients, and this might be explained by a constellation of factors, such as tumour growth, lymph node invasion and progression to local or distant metastases [6, 9]. A recent issue of the US Department of Health and Human Services estimated that malignant breast tumour doubles in size with medians between 45 and 260 days [9]. Unfortunately, our results showed that the average DTis for Brazilian BC patients in the overall period, before and during the COVID-19 pandemic were 122.4, 122.5 and 122.3 days, respectively. Although no differences were observed in the average waiting times for BC treatment onset between periods in our analyses, COVID-19 was associated with a greater chance of relevant delays (OR = 1.043; *p* < 0.05), and this controversial finding may be the result of differences in the number of patients in the sample sets before and during the pandemic years. We believe that the small number of cases in the patient collective assembled in 2020–2021 (*n* = 64,284) compared to the sample set gathered in the pre-COVID-19 years (*n* = 251,667) may have interfered with the DTi interval averages and standard deviations and could explain the lack of DTi intervals differences between these periods.

The regression analyses showed an upward trend in the number of BC treatments offered in the pre-pandemic years, which highlights that Brazilian public health was increasingly supplying treatments for newly diagnosed BC patients (growth = 7.0%/year); yet, our results evinced that COVID-19 imposed a drastic reduction in the volume of treatments that were being provided by the SUS. Furthermore, we found a significantly greater chance of Brazilian patients starting BC treatment with an interval longer than 60 days during the COVID-19 pandemic, and this might be related to also the upward trend found in the group of patients with delayed treatment onset in the pre-COVID-19 years.

Brazil is a considerably large country with a continental dimension, and this wide territorial area may be the root of inequalities in the healthcare structure between regions [3, 27]. South Brazil presents a remarkably high incidence of BC [1, 5], and this region exhibited the lowest percentages of patients with delayed treatment initiation in our analyses, even when facing COVID-19. The Southeast showed stability in the percentage of patients with delayed treatment onset between the two periods.

The South of Brazil displays the highest Basic Education Development Index (IDEB = 6.17), the highest per capita income (US$ 320.73), the highest percentage of working people with formal employment (70.6%) and the highest Human Development Index (HDI = 0.756), and similarities are found in the Southeast (IDEB = 6.03; per capita income = US$ 299.40; formal employment = 65.1%; and HDI = 0.754) [27]. These demographic features indicate a proper socioeconomic development of these regions, which might reflect a better public health structure, and it is relevant for our analysis since less access to health care is associated with socioeconomic vulnerabilities in Brazil [3, 8, 27].

The North and the Northeast exhibit the worst sociodemographic indicators in Brazil (N: IDEB = 5.29; per capita income = US$ 176.71; formal employment = 45.4%; HDI = 0.684; NE: IDEB = 5.12; per capita income = US$ 163.48; formal employment = 40.7%; and HDI = 0.660) [27], which may explain why these regions held the highest proportions of patients with delayed treatment. The Midwest displays demographic indicators that suggest adequate regional development (IDEB = 5.73; per capita income = US$ 261.17; formal employment = 59.1%; and HDI = 0.730) [27], yet, this region experienced the worst impact on delays for BC treatment onset during the COVID-19 outbreak, with the greatest percentage increase between periods (+7.86%).

Male cases computed only 0.7% of our sample, however, men were older and exhibited longer waiting times. Moreover, women began to exhibit a higher frequency of advanced BC diagnoses with the arrival of COVID-19 in Brazil, yet significant chances of men displaying advanced-stage disease at diagnosis more often during the pandemic were not observed since these men had already been bearing a higher proportion of advanced disease even before COVID-19.

Some reports have implied that male patients are more frequently diagnosed with advanced BC and have a worse prognosis due to poor awareness that BC can affect men, lack of attention by men (and even by health professionals) at breast symptoms onset, the experience of embarrassment at breast symptom onset, and all these factors combined may result in delays in male BC care [19, 28, 29]. A retrospective study in Hong Kong (1998–2018) showed that the average interval between symptom onset and the first consultation for 56 men with BC was 12.4 months, which could reach up to 120 months [29]. Researchers have demonstrated little interest in publishing about the occurrence of BC in men, yet male BC incidence appears to be slowly rising in developing countries in the last decades [28], and BC incidence in Brazilian men tripled from 1998 to 2008 [19]. Our results showed that the number of newly-diagnosed male BC treatments provided annually increased by 3.64% per year between 2013 and 2021, which, in turn, also reflects an increase in the BC incidence in Brazilian men from our sample.

As aforementioned, the proportion of advanced-stage disease in female BC diagnosis increased with the arrival of COVID-19 in Brazil and imposed a 34.4% higher chance of diagnosing advanced disease, yet early-stage patients exhibited a greater chance of delayed treatment onset during and even before the pandemic, which places women with potentially curable diseases at risk for upstaging and worse outcomes. Lengthy time to treatment initiation is often associated with the risk of BC upstaging in developing countries, even to a status of incurable disease [3, 9].

A report on the impact of the COVID-19 pandemic on BC in São Paulo/Brazil also observed an increment in the proportion of advanced-stage disease during the pandemic (2019–2020: early/advanced = 72.9%/36.1%; 2020–2021: early/advanced = 47.0%/53.0%) [25]. These observations might be explained by the ‘stay-at-home’ strategies initially adopted by Brazilian state governments in an attempt to restrain the transmission of the coronavirus, for instance, the lockdown policy, the lower circulation of non-COVID-19 patients in healthcare services and the postponement of screening tests, which, ultimately, may have delayed patient evaluation and led to more advanced-stage presentations at diagnosis [23–25, 30].

At the time of writing, no previous nationwide study statistically evaluated the effects of COVID-19 on the waiting time for BC treatment onset in Brazil and its regions and assessed the impact of the pandemic on BC staging and procedures. The time interval to the first cancer treatment after a confirmed diagnosis is used as a quality-of-service parameter in many healthcare centres worldwide [9, 16, 31, 32]. In our investigation, none of the treatment options fully complied with the 60-Day Law, and, despite the surgical modality having computed better waiting times, our diagnosis-to-surgery intervals might be sufficient to impact the survival of Brazilian patients with BC. An 8,860-patient retrospective study with BC cases in the US (1997–2006) showed an 80% 5-year survival in women with a diagnosis-to-surgery interval longer than 42 days, which was considerably lower than the 90% 5-year survival in those who underwent surgery in less than 14 days after diagnostic confirmation [32].

Our patients waited an overall average of 82 days to undergo surgery, however, the averages were 84 and 70 days before and during the COVID-19 pandemic, respectively. In early 2020, the American College of Surgeons (ACS) [33] initially recommended the immediate suspension of elective procedures or, at least, surgeons should curtail the performance of elective surgeries in the face of the COVID-19 spread worldwide. The Brazilian College of Surgeons and the Brazilian Society of Oncological Surgery soon adopted the ACS recommendation and instructed a reduction in the volume of elective surgeries nationwide, including oncological surgeries, although reinforced the need for a case-by-case evaluation [33, 34]. Later 2020, the Brazilian Ministry of Health announced the resumption of oncological surgeries in the SUS routine with the premise that ‘cancer patients cannot wait’ [34]. In light of these events, Bonadio et al. [25] hypothesised that non-oncological patients were not referred to surgical centres during the periods of restrictions, facilitating the access of cancer patients to surgery and shortening the diagnosis-to-surgery interval. Nevertheless, our analyses have shown a significant reduction in the number of BC surgeries performed during COVID-19.

Our data also implied that one in almost every five patients who had surgery as the first treatment modality of choice exhibited a 0-day DTi. This finding suggests that this patient underwent surgery on the same day that received the diagnosis confirmation of BC, which does not represent the most common approach to invasive malignant tumours [9]. Conversely, the 0-day DTi may also reflect a misapplication of codes on public health system reporting forms that feed the database, either from the ICD-10 or the SUS procedures list, which, ultimately, have biased our data analyses regarding surgery. Furthermore, radiotherapy as a first treatment for BC is unusual and accounted for only 6.7% of first treatments in our sample. We believe that these are patients for whom surgery was not immediately available, and radiotherapy enabled better control of critical symptoms, such as local haemostasis.

Limitations exist within the scope of this investigation and including the retrospective and observational design of the study and the database, whose structure did not allow us to evaluate patients regarding ethnicity, level of education, per capita income, access to health insurance, history of BC or other malignancy, the first and the number of symptoms noticed, the circumstance of BC detection and referral source. Besides, the database did not provide the number of cases with ‘missing data’ by year and by gender, so it was not possible to measure whether there were temporal or gender differences in the completion of data in our investigation. The database did not examine information about the initial staging at diagnosis and the reassessment for upstaging after a long waiting time for treatment initiation. Clinical staging of BC in the Brazilian public health practice follows the classification system established by the AJCC; however, the platform did not disclose if the reports complied with the AJCC staging system changes for BC stated in 2018. Further, it was not possible to differentiate the intention of chemotherapy as palliative or neoadjuvant, nor to evaluate tumour size, angiolymphatic or perineural invasion, histologic grade, molecular subtypes and laterality. Therefore, the present study could not associate these features with delays in BC treatment onset during COVID-19, which, in turn, constitutes grounds for performing more robust, multicentre and primary studies.

## Conclusion

In conclusion, our results show that despite Federal Law No. 12,732/2012 completing 10 years in operation in 2022, most BC patients still initiate treatment with delays longer than 60 days after the diagnostic confirmation, especially if the presentation is with early-stage disease, advanced age or the patient is a man. Our time series analysis indicated that the number of patients with delayed treatment onset has been predominant since 2014, with increasing trends throughout the years, and the panorama worsened with the arrival of COVID-19 in Brazil when the odds of detecting metastases and the frequency of advanced disease outnumbered early-stage disease at diagnosis. The ideal prospect to improve the BC mortality burden in post-COVID-19 Brazil requires significant investments in cancer care nationwide, effective resumption of BC screening programs, and the development of public policies for educating men and women about BC diagnosis and the importance of timely presentation and treatment. Undoubtedly, the 60-Day Law is a milestone in the history of Brazilian public health as an effort to reduce health system delays to cancer treatment initiation, but surveillance still needs to be improved to ensure that this law is properly implemented.

## Conflicts of interest

There is no conflict of interest

## Funding

None

## Author contributions

Conception and design: All authors. Collection and assembly of data: JHFN and CNS. Data analysis and interpretation: All authors. Manuscript writing: JHFN. Revised the language/article: All authors. Final approval of manuscript: All authors.

## Figures and Tables

**Figure 2.**
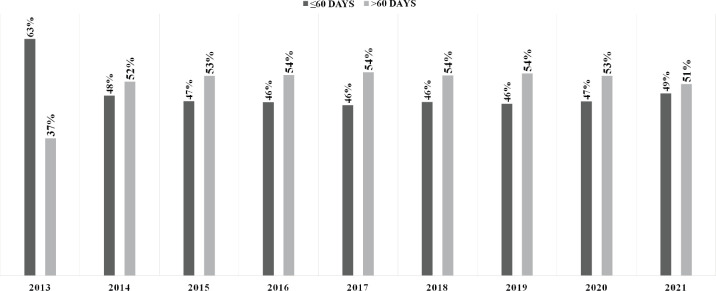
Frequency distributions of time intervals between BC (C.50) diagnosis and treatment initiation in Brazil.

**Table 1.** Number of patients initiating BC (C.50) treatment in Brazil, by gender and time intervals.

	Total population				Females				Males			
	Total	≤60 days	>60 days	Missing data	Total	≤60 days	>60 days	Missing data	Total	≤60 days	>60 days	Missing data
2013	29,204	18,478	10,726	-	29,060	18,376	10,684	-	144	102	42	-
2014	33,011	15,889	17,122	-	32,802	15,788	17,014	-	209	101	108	-
2015	33,640	15,683	17,957	-	33,408	15,577	17,831	-	232	106	126	-
2016	35,193	16,312	18,881	-	34,945	16,211	18,734	-	248	101	147	-
2017	36,701	16,739	19,962	-	36,455	16,625	19,830	-	246	114	132	-
2018	40,344	18,742	21,602	-	40,041	18,583	21,458	-	303	159	144	-
2019	43,574	20,028	23,546	-	43,242	19,849	23,393	-	332	179	153	-
2020	41,023	19,117	21,906	-	40,754	18,981	21,773	-	269	136	133	-
2021	23,261	11,342	11,919	-	23,118	11,261	11,857	-	143	81	62	-
**Total (%)**	**315,951 (100.0%)**	**152,330 (48.2%)**	**163,621 (51.8%)**	**46,077 (-)**	**313,825 (100.0%)**	**151,251 (48.2%)**	**162,574 (51.8%)**	**43,400 (-)**	**2,126 (100.0%)**	**1,079 (50.8%)**	**1,047 (49.2%)**	**2,677 (-)**
Average	35,106	16,926	18,180	-	34,869	16,806	18,064	-	236	120	116	-
SD	6,309	2,606	4,387	-	6,247	2,581	4,350	-	64	32	39	-
Median	35,193	16,739	18,881	-	34,945	16,625	18,734	-	246	106	132	-
IQR: Q1	33,011	15,889	17,122	-	32,802	15,788	17,014	-	209	101	108	-
IQR: Q3	40,344	18,742	21,602	-	40,041	18,583	21,458	-	269	136	144	-

SD: Standard deviation; IQR: Interquartile range

**Table 2.** DTi averages in days for BC (C.50), by gender and time intervals.

	Total period (2013–2021)		Pre-COVID-19 (2013–2019)		COVID-19 (2020–2021)	
	Average	SD	Average	SD	Average	SD
Total population						
Overall	122.4	169.34	122.5	166.30	122.3	180.12
≤60 days	28.8	19.02	28.7	18.98	28.8	19.17
>60 days	197.7	196.75	196.3	191.78	203.0	214.68
Females						
Overall	122.3	168.94	122.3	165.93	122.1	179.60
≤60 days	28.8	19.00	28.8	18.96	28.9	19.14
>60 days	197.4	196.25	196.0	191.33	202.6	214.00
Males						
Overall	143.3	220.29	142.9	212.70	144.8	247.51
≤60 days	22.4	20.59	23.7	20.47	18.0	20.36
>60 days	248.0	258.03	240.9	246.60	278.8	301.01

SD: Standard deviation

**Table 3.** Average age, peak age and peak age group in years old of patients initiating treatment.

	Total period (2013–2021)				Pre-COVID-19 (2013–2019)				COVID-19 (2020–2021)			
	Average	SD	Peak age	Peak age group	Average	SD	Peak age	Peak age group	Average	SD	Peak age	Peak age group
Total population												
Overall	56.6	15.22	50	50–54	56.7	15.19	50	50–54	56.4	15.29	54	55–59
≤60 days	55.6	15.44	50	50–54	55.8	15.41	49	50–54	55.1	15.56	54	50–54
>60 days	57.4	14.75	50	50–54	57.5	14.49	50	50–54	57.5	14.96	60	55–59
Females												
Overall	56.6	15.19	50	50–54	56.6	15.17	50	50–54	56.4	15.28	54	55–59
≤60 days	55.6	15.42	50	50–54	55.7	15.39	49	50–54	55.1	15.55	54	50–54
>60 days	57.2	14.22	50	50–54	57.4	14.92	50	50–54	57.5	14.94	60	55–59
Males												
Overall	63.6	16.27	68	65–69	63.9	16.59	68	65–69	62.7	16.45	69	65–69
≤60 days	62.3	14.60	58	65–69	63.8	16.76	58	65–69	61.2	15.97	69	65–69
>60 days	63.9	16.19	68	65–69	64.0	16.41	68	65–69	64.5	16.80	71	65–69

SD: Standard deviation

**Table 4.** Age comparison for the chances of waiting longer than 60 days for treatment initiation.

	Total period (2013–2021)			Pre-COVID-19 (2013–2019)			COVID-19 (2020–2021)		
	OR	CI 95%	*p*-value	OR	CI 95%	*p*-value	OR	CI 95%	*p*-value
<50 years	1.0^a^	***-***	***-***	1.0^a^	***-***	***-***	1.0^a^	***-***	***-***
50–59 years	1.20	1.184–1.226	<0.05	1.19	1.170–1.217	<0.05	1.24	1.197–1.286	<0.05
60–69 years	1.32	1.298–1.346	<0.05	1.29	1.268–1.322	<0.05	1.41	1.361–1.469	<0.05
70–79 years	1.32	1.299–1.359	<0.05	1.30	1.266–1.334	<0.05	1.42	1.361–1.496	<0.05
80 years and over	1.28	1.245–1.332	<0.05	1.25	1.207–1.303	<0.05	1.40	1.310–1.506	<0.05

a Reference

**Table 5.** Clinical staging of patients initiating BC (C.50) treatment, by gender and time intervals.

	Total population		≤60 days group		>60 days group		Males		Females	
	*N*	%	*N*	%	*N*	%	*N*	%	*N*	%
Total period (2013–2021)										
I	44,316	21.92%	12,597	15.79%	31,719	25.92%	175	13.93%	44,141	21.97%
II	64,918	32.11%	20,706	25.96%	44,212	36.13%	358	28.50%	64,560	32.14%
III	64,950	32.13%	32,296	40.49%	32,654	26.68%	455	36.23%	64,495	32.10%
IV	27,961	13.83%	14,173	17.77%	13,788	11.27%	268	21.34%	27,693	13.79%
Total	202,145	100.00%	79,772	100.00%	122,373	100.00%	1,256	100.00%	200,889	100.00%
Missing data	113,806	-	72,558	-	41,248	-	870	-	112,936	-
Pre-COVID-19 (2013–2019)										
I	35,394	22.93%	10,058	16.82%	25,336	26.79%	148	14.67%	35,246	22.98%
II	50,716	32.85%	15,852	26.51%	34,864	36.86%	288	28.54%	50,428	32.88%
III	47,335	30.66%	23,198	38.80%	24,137	25.52%	361	35.78%	46,974	30.63%
IV	20,935	13.56%	10,686	17.87%	10,249	10.84%	212	21.01%	20,723	13.51%
Total	154,380	100.00%	59,794	100.00%	94,586	100.00%	1,009	100.00%	153,371	100.00%
Missing data	97,287	-	62,077	-	35,210	-	705	-	96,582	-
COVID-19 (2020–2021)										
I	8,922	18.68%	2,539	12.71%	6,383	22.97%	27	10.93%	8,895	18.72%
II	14,202	29.73%	4,854	24.30%	9,348	33.64%	70	28.34%	14,132	29.74%
III	17,615	36.88%	9,098	45.54%	8,517	30.65%	94	38.06%	17,521	36.87%
IV	7,026	14.71%	3,487	17.45%	3,539	12.74%	56	22.67%	6,970	14.67%
Total	47,765	100.00%	19,978	100.00%	27,787	100.00%	247	100.00%	47,518	100.00%
Missing data	16,519	-	10,481	-	6,038	-	165	-	16,354	-

N: Number of cases

**Table 6.** Annual average numbers of patients initiating BC (C.50) treatment in Brazil, by staging and gender.

	Pre-COVID-2019 (2013–2019)		COVID-2019 (2020–2021)	
	Average	SD	Average	SD
Total population				
Stage I	5,056	789	4,461	1,867
Stage II	7,245	1,153	7,101	2,193
Stage III	6,762	1,354	8,808	1,675
Stage IV	2,991	589	3,513	901
Total (Stages I–IV)	35,952	4,797	32,142	12,560
Females				
Stage I	5,035	787	4,448	1,860
Stage II	7,204	1,142	7,066	2,179
Stage III	6,711	1,349	8,761	1,668
Stage IV	2,960	578	3,485	884
Total (Stages I–IV)	35,708	4,737	31,936	12,471
Males				
Stage I	21	5	14	6
Stage II	41	11	35	14
Stage III	52	11	47	7
Stage IV	30	11	28	17
Total (Stages I–IV)	245	61	206	89

SD: Standard deviation

**Table 7.** Frequency distribution of treatment modalities by time intervals for BC (C.50) treatment initiation.

DTi	Surgery	Chemotherapy	Radiotherapy	Other
0-day interval	19.6%	2.9%	2.3%	3.3%
1–10 days	3.0%	2.7%	1.3%	2.0%
11–20 days	5.1%	4.9%	1.9%	3.5%
21–30 days	7.3%	7.3%	3.1%	3.8%
31–40 days	7.6%	8.0%	4.0%	6.4%
41–50 days	8.2%	8.4%	4.8%	6.1%
51–60 days	6.8%	7.0%	4.9%	6.9%
61–90 days	15.6%	17.3%	14.9%	16.0%
91–120 days	9.4%	12.1%	12.2%	13.4%
121–300 days	14.1%	22.2%	35.9%	32.8%
301–365 days	1.3%	1.9%	6.3%	2.0%
366–730 days	1.5%	3.0%	7.2%	3.0%
More than 2 years	0.5%	2.3%	1.1%	0.8%

DTi: diagnosis-to-treatment interval

**Table 8.** DTi averages in days for BC (C.50) treatment initiation, by treatment modalities.

	Average DTi	SD
Total period (2013–2021)		
Surgery	82.0	117.1
Chemotherapy	133.8	184.81
Radiotherapy	179.4	167.62
Pre-COVID-2019 (2013–2019)		
Surgery	84.1	116.5
Chemotherapy	134.9	182.5
Radiotherapy	173.8	168.0
COVID-2019 (2020–2021)		
Surgery	70.8	119.5
Chemotherapy	130.6	192.0
Radiotherapy	205.5	163.5

DTi: Diagnosis-to-treatment interval; SD: Standard deviation

**Table 9.** Treatment modalities comparison for the chances of waiting longer than 60 days for treatment initiation.

	OR	CI 95%	p-value
Total period (2013–2021)			
Surgery	1.0^a^	-	-
Chemotherapy	2.78	2.738–2.826	<0.05
Radiotherapy	6.75	6.522–6.990	<0.05
COVID-19 (2020–2021) versus pre-COVID-19 (2013–2019) years			
Surgery	0.94	0.907–0.977	<0.05
Chemotherapy	0.87	0.855–0.892	<0.05
Radiotherapy	1.97	1.790–2.181	<0.05

a Reference

NS: Not significant

**Table 10.** Annual average number of treatment modalities on patients initiating BC (C.50) treatment, by time intervals.

	Pre-COVID-2019 (2013–2019)		COVID-2019 (2020–2021)	
	Average	SD	Average	SD
Total population				
Total	35,952	4,797	32,142	12,560
Surgery	12,833	967	7,347	5,611
Chemotherapy	20,545	3,804	22,877	6,005
Radiotherapy	2,503	169	1,868	908
≤60 days				
Total	17,410	1,669	15,230	5,498
Surgery	8,465	867	4,944	3,549
Chemotherapy	8,314	969	10,011	1,848
Radiotherapy	607	163	261	87
>60 days				
Total	18,542	4,082	16,913	7,062
Surgery	4,367	755	2,403	2,062
Chemotherapy	12,231	3,072	12,866	4,158
Radiotherapy	1,896	324	1,608	821

SD: Standard deviation
